# Cladribine effects on patient-reported outcomes and their clinical and biometric correlates in highly active relapsing multiple sclerosis at first switch: the observational, multicenter, prospective, phase IV CLADFIT-MS study

**DOI:** 10.3389/fneur.2024.1422078

**Published:** 2024-07-18

**Authors:** Giovanna Borriello, Clara Grazia Chisari, Davide Maimone, Massimiliano Mirabella, Damiano Paolicelli, Francesco Assogna, Sandro Caradonna, Francesco Patti

**Affiliations:** ^1^Multiple Sclerosis Center, San Pietro Fatebenefratelli Hospital, Rome, Italy; ^2^Department of Public Health, Federico II University, Naples, Italy; ^3^Department of Medical and Surgical Sciences and Advanced Technologies, GF Ingrassia, University of Catania, Catania, Italy; ^4^UOS Multiple Sclerosis, AOU Policlinico “G Rodolico-San Marco”, University of Catania, Catania, Italy; ^5^Centro Sclerosi Multipla, UOC Neurologia, Azienda Ospedaliera per l’Emergenza Cannizzaro, Catania, Italy; ^6^Multiple Sclerosis Center, Fondazione Policlinico Universitario “A. Gemelli” IRCCS, Rome, Italy; ^7^Centro di Ricerca per la Sclerosi Multipla “Anna Paola Batocchi”, Università Cattolica del Sacro Cuore, Rome, Italy; ^8^Neurology Unit, Department of Translational Biomedicine and Neuroscience (DiBraiN), Policlinico General Hospital, University of Study of Bari, Bari, Italy; ^9^Merck Serono S.p.A. Italy, An Affiliate of Merck KGaA, Rome, Italy

**Keywords:** relapsing–remitting multiple sclerosis, disease-modifying treatment, cladribine tablets, observational study, patient-reported outcomes, wearable devices, CLADFIT study

## Abstract

Patient-reported outcomes (PROs) are essential for understanding the effects of MS and its treatments on patients’ lives; they play an important role in multiple sclerosis (MS) research and practice. We present the protocol for an observational study to prospectively assess the effect of cladribine tablets on PROs and their correlation to disability and physical activity in adults with highly active relapsing MS switching from a first disease modifying drug (DMD) to cladribine tablets in routine clinical practice at study sites in Italy. The primary objective will be to evaluate changes from baseline in the impact of highly active MS on self-assessed physical functioning 52 weeks after the switch to cladribine tablets using the Multiple Sclerosis Impact Scale-29 (MSIS-29). Secondary objectives will include self-assessed psychological impact of highly active MS in daily life and general health after the switch to cladribine tablets as well as changes in cognitive function, anxiety, and depression symptoms. Additional PRO measures will include the Hospital Anxiety and Depression Scale (HADS), the EuroQoL 5-Dimension 5-Level (EQ-5D-5L), the Work Productivity and Activity Impairment Questionnaire: Multiple Sclerosis (WPAI:MS), and the Patient-Reported Outcomes Measurement Information System (PROMIS). Wearable devices will acquire activity data (step counts, walking speed, time asleep, and energy expenditure). Additional clinical, radiological, and laboratory data will be collected when available during routine management. The findings will complement data from controlled trials by providing insight from daily clinical practice into the effect of cladribine tablets on the patient’s experience and self-assessed impact of treatment on daily life.

## 1 Background

Multiple sclerosis (MS) is a chronic, autoimmune, inflammatory disease of the central nervous system (CNS) which attacks myelinated axons, destroying, to varying degrees, both myelin and axonal tissue (1–3). The disorder, which affects an estimated 137,000 patients in Italy (4), typically presents between the ages of 20 and 45 years, being less frequent in childhood or late middle age (5). The cause of MS is unknown, but it appears to involve a combination of genetic susceptibility and non-genetic triggers, such as viral, metabolic, or environmental factors, that result in a self-sustaining autoimmune disorder which leads to recurrent immune attacks on the CNS (5–7).

The course of MS is highly varied and is often characterized initially by episodes of reversible neurological deficits that are followed by progressive neurological deterioration over time (8). Some patients experience a highly active disease course, with rapid and early-onset disability, heralded by high relapse rates and early motor, cerebellar and/or cognitive dysfunction (9). Highly active relapsing MS (RMS) is characterized by neurological deterioration causing motor and cognitive dysfunction that impacts health-related quality of life (HRQoL) (10). Despite the recent introduction of new disease-modifying therapies (DMTs) for treating MS (11–13), a substantial clinical burden remains, and new approaches are required to reduce the number of relapses and prevent disability progression, while maintaining QoL (14).

Cladribine is a synthetic deoxyadenosine analog prodrug that is activated through phosphorylation by deoxycytidine kinase that preferentially accumulates to cytotoxic levels in lymphocytes. This results in targeted and sustained reductions of the T and B lymphocytes implicated in MS pathogenesis (15). Cladribine tablets were approved for the treatment of highly active RMS in adults by the European Medicines Agency (EMA) in 2017 (16).

Two short courses of cladribine tablets across 2 consecutive years (cumulative dose 3.5 mg/kg) provides clinically and statistically significant benefits in patients across the RMS spectrum (early to late stages, treatment naïve, or experienced patients) (17, 18). Each treatment course consists of 2 treatment weeks, one at the beginning of the first month and one at the beginning of the second month of the respective treatment year. In the approved treatment regimen, these short treatment courses in years 1 and 2 are followed by 2 years without treatment (16).

Patients with MS frequently report limitations in daily activities due to fatigue, problems with balance or coordination, cognitive impairment, and sleep disturbances (19). In addition, MS has been associated with diminished QoL due to the limitations it may impose on employment and social activities (20, 21). Improving QoL is therefore an important goal in the treatment of MS and QoL assessment can help guide healthcare decisions toward this end (22).

Patient-reported outcomes (PROs) play an increasing role in MS research and practice and are essential toward understanding the effects of MS and its treatment on patients’ lives. For MS, the relevance of QoL as an overall subjective measure is underpinned by studies showing that increase in QoL during treatment is accompanied by improvement in fatigue, depression, or cognition (23). In the CLARITY trial, in addition to the observed reduction in relapse, disability progression, and Magnetic Resonance Imaging (MRI)-assessed disease activity compared with placebo, there was a significant improvement in EuroQoL 5-Dimension (EQ-5D) index scores and a trend toward improvement as measured via the Multiple Sclerosis Quality of Life-54 (MSQoL-54) Questionnaire (24). The ongoing open label, single-arm, Phase IV CLARIFY-MS trial (NCT03369665) expanded these findings, showing statistically significant improvements over 24 months in the physical and mental health composite scores of the MSQoL-54 (25).

Patient-reported outcome assessment is expected to have an increasing role in the future. Although it is important to evaluate traditional measures, e.g., relapse count and disability progression, such information alone is insufficient toward the comprehensive assessment of disease outcomes; instead, the use of PROs and QoL assessments that address the physical, psychosocial, and sexual aspects of the disease (26) should be undertaken. The application of tools such as the MSIS-29 (27) and the Patient-Reported Outcomes Measurement Information System (PROMIS) (28) can overcome the deficiencies of traditional assessments [e.g., the Expanded Disability Status Scale (EDSS) (29), and MRI findings] by capturing outcomes that are most important to MS patients in terms of improvement in daily life and experience (30).

The fluctuating nature of MS symptoms, fatigue during the course of the day or presence of relapse, for example, can make it difficult to obtain a representative measurement of a patient’s condition at a clinical visit. Toward this end, the use of wearable technology allows continuous monitoring of function during the patient’s normal daily routine (31, 32), thereby allowing their disease status to be measured and monitored over time (33).

Multiple sclerosis symptoms include motor impairment and gait disturbances (34, 35), and patients with MS have been found to be less physically active compared with the general population (36, 37). Mobility measures based on functional performance, physical assessment, and patient self-reporting, often have low sensitivity. Wearable devices with inertial measurement units help to overcome such limitations and can be used to evaluate functional capacity in real-world settings (38). These wearable activity trackers may be affected by gait disturbances in patients with advanced MS, be less accurate than professional instruments when measuring low activity levels (39), but they are suitable for estimating changes in intra-individual activity in patients with mild or moderate MS (40, 41).

There are several therapeutic options for treating RMS, and it is important to identify optimal sequencing, understand the patient journey through the various treatments, and generate evidence to inform therapy switches. In the pivotal CLARITY study [17], approximately one-third of patients had switched to cladribine after treatment with interferon-beta or glatiramer acetate; however, dimethyl fumarate and teriflunomide were not yet approved at that time.

## 2 Aims

The aim of this study will be to evaluate the effect of cladribine tablets on PROs and physical activity, and their correlation with disability, as well as the clinical effectiveness and tolerability of cladribine in patients with highly active RMS who are switching from a first-line MS DMT to cladribine tablets as their first second-line treatment in routine clinical practice. CLADFIT also aims to investigate the reasons for switching to cladribine and provide important information on washout strategies after first-line therapies. This data will be useful to inform therapeutic choices supported by scientific evidence. Quality of life, health utilization and treatment satisfaction data will also support pharmacoeconomic analyses.

## 3 Study objectives

### 3.1 Primary objective

To evaluate changes from baseline in the impact of highly active RMS on self-assessed physical functioning in daily life after the switch to cladribine tablets.

### 3.2 Secondary objectives

To assess the following parameters among patients with highly active RMS after the switch to cladribine tablets:

- Changes in the self-assessed psychological impact of disease burden on daily life;
- Changes in self-assessed general health;
- Changes in cognitive function;
- Changes in self-assessed anxiety and depression symptoms;
- The relationship between changes in PROs (e.g., physical health status, psychological health status, and anxiety and depression symptoms), cognitive function, and/or data from wearable activity trackers;
- The annualized relapse rate over the first and second year of treatment;
- Changes in employment status;
- Real-world pharmacoeconomic data;
- Safety of cladribine tablets in real-world clinical practice.

### 3.3 Other objectives

To assess, among patients with highly active RMS:

- The level of disability in patients after the switch to cladribine tablets;
- Changes in MRI lesions identified on scans performed at the discretion of the treating physician, following routine clinical practice, at any visit after the switch to cladribine tablets;
- The safety of cladribine tablets 24 months after treatment initiation.

## 4 Methods

This protocol complies with the European Network of Centres for Pharmacoepidemiology and Pharmacovigilance (ENCePP) Checklist for Study Protocols (Revision 4). Trial Registration: European Union Electronic Register of Post-Authorisation Studies (EU PAS) number EUPAS43893.

### 4.1 Observational study design

This observational, multicenter, 2-year prospective, phase IV study will be conducted across approximately 30 sites in Italy, in patients with highly active RMS who are switching to cladribine tablets as their first second-line treatment. Cladribine prescribing will follow clinical practice and the decision to prescribe will be independent of the decision to enroll the patient in the study. The Baseline Visit can take place anytime between the decision to switch to cladribine tablets and the start of the washout period of the previous DMT. Patients will attend visits at the sites as per routine practice; visits are expected at Week 0 (i.e., start of cladribine tablet treatment), Week 24, Week 52, Week 76, and Week 104 after starting treatment. At each visit, clinical and radiological assessments will be recorded as part of routine clinical practice and will be complemented by PROs.

The PROs will include the MSIS-29 (27), the Hospital Anxiety and Depression Scale (HADS) (42), the EuroQoL 5-Dimension 5-Level (EQ-5D-5L) (43, 44), the Work Productivity and Activity Impairment Questionnaire: Multiple Sclerosis (WPAI:MS) (45), and PROMIS (28). Patients will be relatively early in the (highly active) RMS disease course; therefore, this study will include activity tracking with Fitbit® devices to record physical activity levels (range of movement, step counts, walking speed, heart rate, energy expenditure and time spent asleep). Correlations between these activity parameters and changes in PROs after switching to cladribine tablets will be evaluated. The validated Italian versions of the PRO scales are summarized in Table 1.

**Table 1:** Patient reported outcome (PRO) instruments and validated Italian versions.

Instrument	Validated Italian version
Multiple Sclerosis Impact Scale-29 (MSIS-29) (27)	MSIS-29, Italy (Italian edition, 22 Feb 13) MAPI Institute (46)
Hospital Anxiety and Depression Scale (HADS) (42)	Costantini (47)
EuroQoL 5-Dimension 5-Level (EQ-5D-5L) (43, 44)	Screen Report Italian – Italy Version 1.0 (48)
Work Productivity and Activity Impairment Questionnaire: Multiple Sclerosis (WPAI:MS) (45)	WPAI:MS V2.2 Italian (Italy) (49)
Patient-Reported Outcomes Measurement Information System (PROMIS) (28)	PROMIS® Item Bank v1.0 – Fatigue – Multiple Sclerosis Short Form 8a_Italian 10 September 2020 (50)
Symbol Digit Modalities Test (SDMT) (51)	SDMT-adm-scoring-instructions, Italy (Italian edition, 16 Apr 2018) MAPI Institute. ID060544 / SDMT-adm-scoring-instructions – Italian version (52)

**Figure 1:**
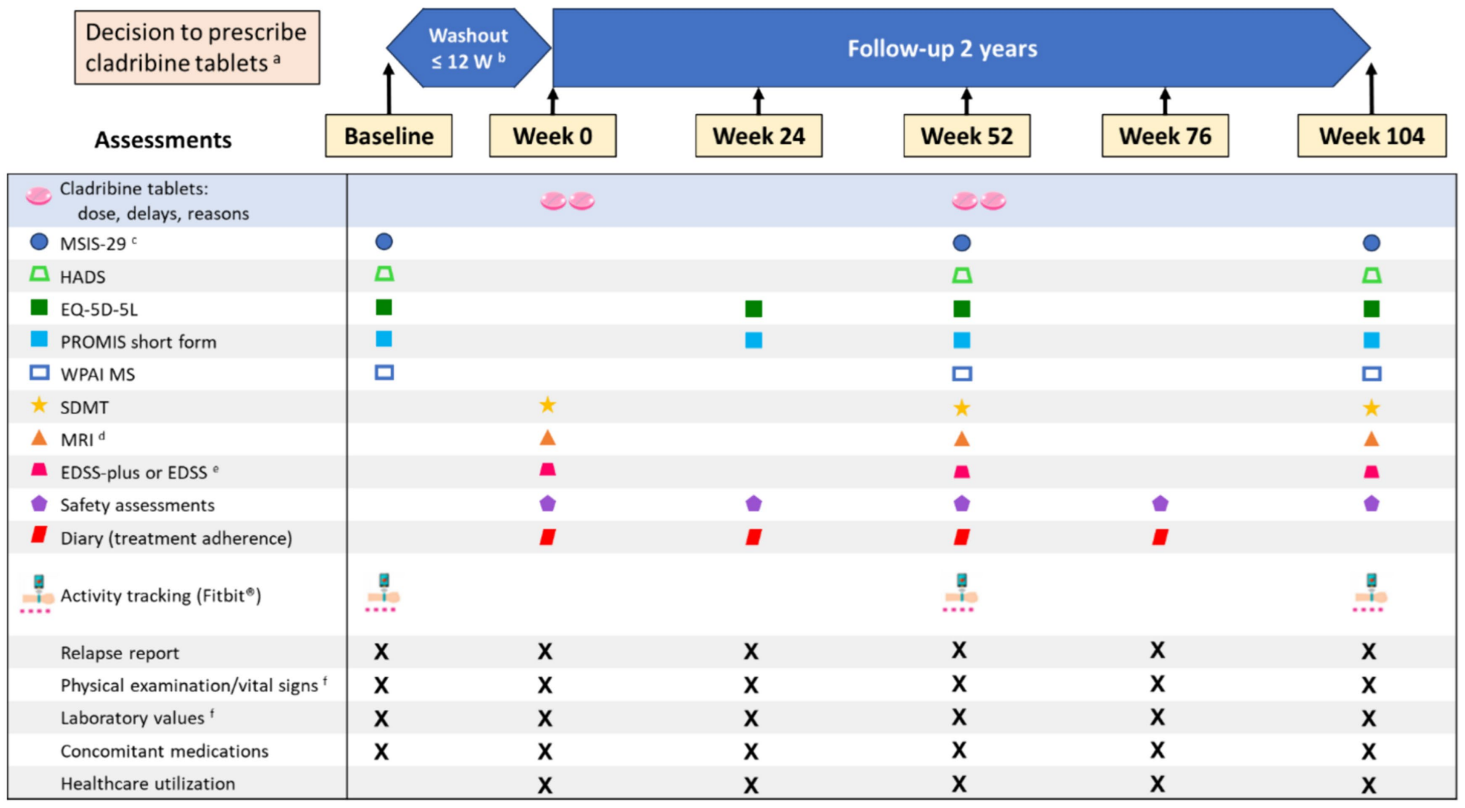
Overview of the CLADFIT study design and visits ^a^ Prescribing according to the SmPC ^b^ If no washout is required, Baseline and Week 0 may be the same visit. ^c^ If the period between Baseline and Week 0 is >4 weeks, the MSIS-29 will be repeated at Week 0. ^d^ MRI results will be collected when available. ^e^ EDSS-plus will be collected if available; otherwise, EDSS will be collected. ^f^ Results from physical examinations, vital signs, and laboratory tests (including lymphocyte counts) will be collected when available. EDSS: Expanded Disability Status Scale; EQ-5D-5L: EuroQoL 5-Dimension 5-Level; HADS: Hospital Anxiety and Depression Scale; MRI: Magnetic Resonance Imaging; MSIS-29: Multiple Sclerosis.

An overview of the study design is presented in Figure 1.

### 4.2 Study population

The study population will be recruited across approximately 30 sites in Italy. Enrolled patients will meet inclusion (Box 1) and exclusion criteria (Box 2); those who do not receive their first dose of cladribine tablets within 12 weeks of Baseline will be withdrawn from the study.

BOX 1 **Inclusion criteria.**

Patients who:

1. Are at least 18 years old;
2. Have highly active RMS* and are switching to cladribine tablets [as per the SmPC (16)] as their first second-line treatment in routine clinical practice;
3. Provide written informed consent to participate and release their personal data.

* Highly active RMS: defined as patients with 1 relapse in the previous year with at least 1 T1 Gd + lesion or at least 9 T2 lesions while receiving other DMDs; or patients with at least 2 relapses in the previous year, regardless of treatment.

DMDs, Disease Modifying Drugs; SmPC, Summary of Product Characteristics.

BOX 2 **Exclusion criteria.**

Patients who:

1. The investigator considers are unable to provide reliable study information, or are likely to be lost to follow-up during The first months of the study;
2. Have contraindications to cladribine tablets according to the SmPC;
3. Are receiving a DMT with an SmPC-defined washout period >12 weeks;
4. Were previously treated with a second-line MS therapy;
5. Have clinically relevant anxiety/depressive disorders that the investigator considers will impede study participation;
6. Are participating in interventional clinical trials.

SmPC: Summary of Product Characteristics.

### 4.3 Sample size

Based on the primary outcome (mean change in the MSIS-29 physical domain score at Week 52), the expected standard deviation of 13 would require 193 patients to obtain a two-sided 95% CI for the mean with a probability of 0.95. Assuming an attrition rate of 10%, 215 patients will be recruited.

### 4.4 Data collection

At the Baseline visit, patients who meet all eligibility criteria and provide informed consent will be enrolled into the study after which baseline data will be collected (e.g., demographic information, medical and medication histories, and comorbidities), including the assessment data shown in Figure 1. When a washout period is not required, Baseline and Week 0 may be the same visit. Patients will attend visits in a routine practice setting scheduled at the discretion of the treating physician; therefore, the indicated visit times are approximate.

### 4.5 Outcome measures

#### 4.5.1 Primary outcome measure

- Change from Baseline in the MSIS-29 physical domain score at Week 52.

The MSIS-29 (27) is a PRO instrument that measures the physical and psychological impact of MS from the patient’s perspective; it includes 20 items in the physical domain and 9 items in the psychological domain. Items explore the impact of MS on the patient’s day-to-day life in the last 2 weeks, either via the patient or their proxy. All items have a 5-point Likert scale response, with options from 1 (‘not at all’) to 5 (‘extremely’). Each domain is scored by summing the responses across items and converting to a 0 to 100 scale, with higher scores indicating greater impact.

#### 4.5.2 Secondary outcome measures

- The MSIS-29 physical, psychological, and combined scores at Baseline, Weeks 52, and 104; the combined MSIS-29 score will be used to represent general health (53);
- Change from Baseline in the MSIS-29 psychological domain score at Weeks 52, and 104;
- Change from Baseline in the MSIS-29 combined score at Weeks 52, and 104;
- Change in cognitive function from Baseline measured by the Symbol Digit Modalities Test (SDMT) at Weeks 52, and 104.

The SDMT (51) is a test of information processing speed; brief and easy to administer, the SDMT has demonstrated remarkable sensitivity in detecting the presence of brain damage as well as changes in cognitive function over time, and in response to treatment. The test consists of 9 abstract symbols, each one being paired with a single digit. The subject is provided with a ‘key’, showing each symbol-digit pair. The subject then views several rows of 9 symbols, which are arranged pseudo-randomly, without the digits, and is asked to voice the digit associated with each symbol as rapidly as possible for 90 s. The single outcome measure (the ‘SDMT’) is the number of correct responses over the 90 s time span, regardless of the number of incorrect responses.

- Change from baseline in anxiety and depression symptoms measured by HADS at Weeks 52, and 104.

The HADS (42) questionnaire is used to detect levels of anxiety and depression in patients, both hospital and community based. The questionnaire includes 14 items (7 on anxiety and 7 on depression), individual scoring for which ranges from 0 to 3, with 3 denoting the highest level of anxiety or depression. Scores include the separate domain scores (anxiety and depression) and the overall score (ranging from 0 to 21 points).

- Change from baseline in general health status measured by EQ-5D-5L at Weeks 24, 52, and 104.

The EQ-5D-5L questionnaire aims to assess health status on a common scale. The EQ-5D-5L essentially consists of 2 pages: the EQ-5D descriptive system and the EQ visual analog scale (EQ-VAS). The descriptive system includes a total of 5 dimensions designated as mobility, self-care, usual activities, pain/discomfort, and anxiety/depression. Each dimension has 5 response levels: no problems, slight problems, moderate problems, severe problems, unable to /extreme problems. The scores obtained from each dimension can be combined into a 5-digit number that describes the subject’s health state, with a higher score indicating a better HRQoL. The EQ-VAS records the subject’s self-rated health status on a vertical visual analog scale, from ‘The best health you can imagine’ to ‘The worst health you can imagine’. The EQ-VAS can be used as a quantitative measure of health outcome that reflects the subject’s own judgment. Both scores will be analyzed in the study.

- Changes in levels of depression, anxiety, fatigue, pain, and physical function, self-assessed via PROMIS between Baseline, and Weeks 24, 52, and 104.

The PROMIS-29 short form questionnaire evaluates 7 health domains: pain, fatigue, depression, anxiety, sleep disturbance, physical function, and social roles (54). Domain scores for depression, anxiety, fatigue, pain, and physical function will be analyzed.

- Evaluation of data (to include range of movement, meters or steps in a day, walking speed, calories burned, heart rate, and hours of sleep) from wearable activity trackers (Fitbit®) over 2 days of observation at Baseline, and Weeks 52, and 104;
- Association between changes in PROs (MSIS-29, HADS), cognitive function (SDMT), and data from wearable activity trackers (Fitbit®), at Baseline, and Weeks 0, 52, and 104;
- Association between levels of depression, anxiety, fatigue, pain, and physical function (self-assessed via PROMIS) and data from wearable activity trackers (Fitbit®) at Baseline, Week 52, and Week 104;
- The annualized rate of clinician-confirmed relapses over the first and second year, respectively, after switching to cladribine tablets;
- Employment status measured by WPAI:MS scores at Baseline, Weeks 52, and 104.

The WPAI:MS assesses work impairment due to MS using 6 questions that yield 4 scores expressed as impairment percentages: absenteeism, presenteeism, work productivity loss, and activity impairment (55). Each of the 4 scores will be analyzed separately.

- Healthcare utilization, recorded separately for inpatient and outpatient resources, including number of hospitalizations (and reasons for), number of hospitalization days, number of emergency room visits, diagnostic or therapeutic procedures, and number of rehabilitation visits, over 2 years following initiation of cladribine tablets;
- Occurrence of adverse drug reactions, adverse events, and serious adverse events during the study period;
- Changes in laboratory values collected in routine care over the study period (Baseline–Week 104).

#### 4.5.3 Other outcome measures

- Disability level measured using the EDSS/EDSS-Plus at Weeks 0, 52, and 104;
- The number of MRI lesions (T2, T1 Gd+, Combined Unique Active (CUA)) identified when, and if, the treating physician requests MRI at Weeks 0, 52, and/or 104.

White matter lesion activity (i.e., new/enlarging T2 lesions, T1 hypointense or Gd-enhancing T1 lesions) and brain atrophy (i.e., percent brain volume change) are valid surrogate endpoints for clinical outcomes (56). As above, MRI scans will be performed at the discretion of the treating physician, following routine clinical practice.

Patients who discontinue treatment will be monitored throughout the follow-up period, according to the study design (Figure 1), regardless of treatment status. A patient will be considered ‘lost to follow-up’ after 3 documented failed attempts at contact; every possible effort will be made to determine the reason for trial discontinuation, reasons for which, when known, will be documented in the electronic case report form.

### 4.6 Data management and statistical methods

Mean change from Baseline to Week 52 (95% CI) will be calculated for MSIS-29 scores in the physical, and psychological domains, and combined. Descriptive statistics will be used for MSIS-29, HADS, WPAI:MS, and SDMT scores at Baseline/Week 0, and Weeks 52, and 104, and for EQ-5D-5L and PROMIS scores at Baseline, Weeks 24, 52, and 104. Healthcare utilization data will also be summarized descriptively. Changes in SDMT, HADS, EQ-5D-5L, WPAI:MS, and PROMIS scores between Baseline/Week 0 and Weeks 52, and 104 will be evaluated using mixed models, taking into account repeated measurements for each patient. Wearable activity tracker data and its relationship with changes in PROs (MSIS-29 or HADS) and cognitive function (SDMT) will also be evaluated using mixed models.

## 5 Discussion

CLADFIT will be subject to several limitations due to its design as an open-label, observational study. Patient assessments will be limited to standard-of-care protocols for study visits, imaging, and laboratory testing. This could result in inconsistent data collection, potentially leading to missing data, information bias, and residual confounding. Interpretation of the results may also be affected by treatment-related variability, such as adherence, discontinuation, or potential interactions with other medications. Additional limitations include potential biases from patient selection, loss to follow-up, and patient recall.

CLADFIT’s strengths include a prospective and longitudinal design, with clear enrolment goals based on the primary objective. Advantages also include a variety of validated outcome measures that assess a broad range of functional, clinical, cognitive, and psychological domains a distinct perspective on the patient experience and the impact of treatment on daily life. Several of the instruments in this study will have overlapping domains, so that psychological function assessed on 5 different instruments, and physical functions on 4 different instruments. Correlations among these results may provide insight into their consistency as a real-world study, CLADFIT will reflect everyday clinical practice more closely than randomized controlled trials by enrolling more representative patient populations and administering treatments that are characteristic of those experienced in routine clinical practice, making its findings useful for assessing and improving patient care.

## Author contributions

GB: Writing – original draft, Writing – review & editing, Data curation, Investigation. CC: Writing – original draft, Writing – review & editing, Data curation, Investigation, Methodology, Validation. DM: Writing – original draft, Writing – review & editing, Data curation, Investigation. MM: Writing – original draft, Writing – review & editing, Data curation, Investigation. DP: Writing – original draft, Writing – review & editing, Data curation, Investigation. FA: Writing – original draft, Writing – review & editing, Conceptualization. SC: Writing – original draft, Writing – review & editing, Conceptualization, Data curation, Formal analysis, Investigation, Methodology, Validation. FP: Writing – original draft, Writing – review & editing, Conceptualization, Data curation, Formal analysis, Investigation, Methodology, Validation.
